# Elevated troponin level as a predictor of inpatient mortality in patients with infective endocarditis in the Southeast United States

**DOI:** 10.1186/s12879-019-4755-z

**Published:** 2020-01-08

**Authors:** William Lorson, Michael P. Veve, Eric Heidel, Mahmoud A. Shorman

**Affiliations:** 1 University of Tennessee Medical Center, Knoxville, TN 37920 USA; 2 University of Tennessee, Graduate School of Medicine, Knoxville, TN 37920 USA; 3 University of Tennessee Health Science Center, Knoxville, TN 37920 USA

**Keywords:** Troponin, Infective endocarditis, Mortality, Injection drug use

## Abstract

**Background:**

Despite recent improvement in management, infective endocarditis (IE) continues to be associated with considerable risk of morbidity and mortality. Early identification of predictors of inpatient mortality is key in improving patient outcomes in IE. The aim of our study was to evaluate the role of serum troponin levels measurements as a marker of increased mortality.

**Methods:**

A case-control study included adult patients with IE admitted to a tertiary care hospital in east Tennessee between December 2012 and July 2017. Cases were defined as patients with definitive IE who died in-hospital; controls were patients who did not die in hospital. First patient admission was included only. Data collected included the patients’ demographic and baseline clinical information, microbiological data, injection drug use status, elevated serum troponins levels.

**Results:**

Two hundred eighty three patients with definitive IE were included; median (IQR) age was 41 (30–57) years, and 153 (54%) patients were men. One-hundred sixty-four (58%) were injection drug users. The most frequent IE type was: 167 (59%) right-sided, 86 (30%) left-sided, 24 (9%) both left and right-sided, and 10 (4%) device related. The most commonly isolated organism was *Staphylococcus aureus* (*n* = 141), and 64% were methicillin-resistant. Two-hundred twelve (75%) patients had a troponin level obtained, and 57 (27%) had an elevated troponin value. Thirty-six (13%) patients died in-hospital; in-hospital mortality was associated elevated troponin values (adjusted odds ratio [adjOR], 7.3; 95%CI, 3.3–15.9), and methicillin-resistant *S. aureus* IE (adjOR 2.6; 95%CI, 1.2–5.8). Forty-four (16%) patients received IE valve surgery, and none of these patients died in the hospital.

**Conclusion:**

Inpatient mortality was higher in patients with IE and elevated cardiac troponin levels compared to patients with normal levels.

## Background

Infective endocarditis (IE) is an infection of the endocardium and heart valves or of a prosthetic valve implant [1]. Great strides have been made in the treatment of IE since Osler’s observation of the disease in the nineteenth century. However, IE continues to be a significant cause of morbidity and mortality [2, 3]. The epidemiology of IE has changed, especially in high-income countries that have observed a significant reduction in IE related to rheumatic disease; while degenerative valvulopathies, prosthetic valve IE (PVE), IE related to cardiovascular implantable electronic devices (CIED) and IE in persons who inject drugs (PWID) have all increased [4, 5]. Presentation with different complications including heart failure, embolic events, abscess formation, conductive disorders, and large vegetation size has been associated with increased in-hospital mortality in patients with IE [6–8].

Troponins are cardiac specific proteins released in case of cardiomyocyte injury. Elevated troponin levels can be a poor predictor of patient’s outcome in many disease states, including coronary artery disease, pulmonary embolism, heart failure, and other cardiac and non-cardiac conditions [9]. There have been some reports with small sample sizes on using troponin as a clinical predictor of mortality in patients with IE [9, 10].

The objective of this study was to describe variables that would predict an increased likelihood of inpatient mortality in patients presenting with IE, and to evaluate the role and usefulness of serum troponin levels measurements as a marker of increased mortality.

## Methods

### Study population

This case-control study included adult patients (aged ≥18 years) with IE admitted to a tertiary care hospital in east Tennessee between December 2012 and July 2017. Data was gathered through retrospective chart review of the electronic medical record. Only the first patient admission for IE was included. The search term “endocarditis” was used on the discharge diagnoses to narrow down the search results. This yielded “endocarditis acute, sub bacterial” and “endocarditis NOS” with ICD 9 codes 421.0 and 424.9, respectively. Patients who met inclusion criteria were confirmed to have definitive IE by the modified Duke Criteria [11].

Data collected included the patients’ demographic and baseline clinical information, microbiological data according to Clinical and Laboratory Standards Institute standards, injection drug use status, hepatitis C status, human immunodeficiency virus (HIV) status, acute renal failure with serum creatinine level of more 2 mg/dl on presentation, requirement of hemodialysis, diabetes mellitus, atrial fibrillation, heart failure, elevated serum troponins levels within 2 days of admission, and severe sepsis/shock during admission. The study was approved by University of Tennessee Graduate School of Medicine institutional review board.

### Outcome measures and key definitions

Cases were defined as patients with definitive IE who died in-hospital; controls were patients who did not die in hospital. Troponin I values were considered elevated if they were ≥ than 0.78 ng/ml as per manufacturers recommendation (Siemens) for critical high, first positive result for the patients were included.

### Statistical analyses

This study was designed to determine risk factors for in-hospital mortality for patients with IE. The exposure of interest was elevated troponin. Descriptive and bivariate analyses were utilized to describe differences in the patient population. Categorical and continuous variables were compared using the Chi-Square, Fisher’s Exact, or Mann-Whitney U-tests. Classification and regression tree (CART) analyses were performed to identify dichotomous breakpoints in continuous variables associated with mortality. Variables associated with in-hospital mortality from bivariate analyses (*P* < 0.2) or deemed clinically relevant a priori were considered for inclusion into a multivariable logistic regression model. Variables were manually entered into the model using a backwards, step-wise approach in order to determine variables independently associated with developing in-hospital mortality while controlling for potential confounders. Other variables were excluded from the model because of unmet clinical or statistical criteria, to preserve the n:k ratio, or to prevent collinearity. The final model was evaluated for goodness-of-fit using the Hosmer-Lemeshow test. All statistics were performed with IBM SPSS Statistics for MacIntosh v.25 (IBM Corp., Armonk, NY).

## Results

Two-hundred and eighty-three patients with IE were included; baseline patient characteristics are listed in Table 1. The median (IQR) age was 41 (30–57) years, and 153 (54%) patients were men. The median (IQR) length of hospital stay was 14 (9–24) days, and 24 (9%) patients left against medical advice. One-hundred sixty-four (58) patients injected illicit drugs, and 110 patients (39%) were positive for hepatitis C virus. The most frequent IE type was: 167 (59%) right-sided, 86 (30%) left-sided, 24 (9%) both left and right-sided, and 10 (4%) device related. Two-hundred twelve (75%) patients had a troponin level obtained, and 57 (27) had an elevated troponin value. Table 2 includes patient characteristics of patients with a troponin value on admission.

**Table 1:** Baseline characteristics of infective endocarditis patients who survived and died

Characteristic *n* (%) or median (IQR)	Total Population *n* = 283	Died in Hospital *n* = 36	Alive *n* = 247	*P*-value
Patient Characteristics				
Age, years	41 (30–57)	46 (33–63)	40 (29–56)	0.14
Sex, male	153 (54%)	24 (67%)	129 (52%)	0.10
Comorbidities				
HIV/AIDS	4 (1%)	0	4 (62%)	1.0
Injection drug use	164 (58%)	24 (67%)	140 (57%)	0.26
Hepatitis C virus	110 (39%)	7 (19%)	103 (42%)	0.01
Diabetes mellitus	40 (14%)	5 (14%)	35 (14%)	0.96
Heart failure	64 (23%)	8 (22%)	56 (23%)	1.0
Atrial fibrillation	31 (110%)	7 (19%)	24 (10%)	0.09
Hemodialysis	15 (5%)	0	15 (6%)	0.23
Acute renal failure/hemodialysis	99 (35%)	27 (75%)	72 (29%)	< 0.001
Septic shock	93 (33%)	28 (78%)	65 (26%)	< 0.001
Troponin obtained	212 (75%)	33 (92%)	179 (73%)	0.01
Infection Characteristics and Outcomes				
Septic Emboli	151 (54%)	27 (75%)	124 (50%)	0.005
Left-sided IE	86 (30%)	18 (50%)	68 (28%)	0.01
IE Valve Surgery	44 (16%)	0	44 (18%)	0.01
MRSA IE	91 (32%)	16 (44%)	75 (30%)	0.09
LAMA	24 (9%)	0	24 (10%)	0.05

**Table 2:** Characteristics of infective endocarditis patients with troponin levels

Characteristic *n* (%) or median (IQR)	Elevated Troponin Level *n* = 57	Normal Troponin level *n* = 155	*P*-value
Patient Characteristics			
Age, years	44 (30–59)	40 (30–55)	0.5
Sex, male	27 (47%)	74 (48%)	1.0
Comorbidities			
Injection Drug Use	34 (60%)	90 (58%)	0.84
Hepatitis C virus	14 (25%)	74 (48%)	< 0.001
Diabetes mellitus	7 (12%)	22 (14%)	0.72
Heart failure	17 (30%)	17 (30%)	0.34
Atrial fibrillation	5 (9%)	21 (14%)	0.35
Acute Renal failure/Hemodialysis	30 (53%)	50 (32%)	0.007
Sepsis/septic shock	40 (70%)	43 (28%)	< 0.001
Infection Characteristics and Outcomes			
Septic emboli	49 (86%)	69 (45%)	< 0.001
Left-sided IE	31 (54%)	37 (24%)	< 0.001
Valve surgery	16 (28%)	17 (11%)	0.002
Died in-hospital	19 (33%)	14 (9%)	< 0.001

Two-hundred eighty-one organisms were isolated from 249 (88%) patients; 34 (12%) patients had culture negative IE. The most commonly isolated aggregate organisms were 91 (32%) methicillin-resistant *Staphylococcus aureus*, 50 (18%) methicillin-sensitive *S. aureus*, 38 (14%) *Enterococcus* spp., 26 (9%) *Streptococcus* spp., 17 (6%) *Pseudomonas aeruginosa*, 5 (2%) other Gram-negatives (*Klebsiella* spp., *Enterobacter* spp., and *Serratia* spp.), and 19 (7%) other organisms.

Thirty-six (13%) patients died in-hospital due to any cause. The results of bivariate analyses and clinical rationale dictated the variables selected for inclusion into a multivariable regression model (Table 3). Variables associated with in-hospital mortality were elevated troponin (adjusted odds ratio [adjOR], 7.3; 95%CI, 3.3–15.9), and MRSA IE (adjOR 2.6; 95%CI, 1.2–5.8). Variables identified to significantly covary with elevated troponin values were: acute renal dysfunction, left-sided endocarditis, severe sepsis/shock, and septic emboli. Hepatitis C virus was excluded as a variable of interest due to the lack of causal inference related to this association. Significant collinearity (*P* < 0.001) was identified between elevated troponin levels and atrial fibrillation, severe sepsis/shock, acute renal failure, left-sided IE, and septic emboli, which ultimately precluded inclusion of these variables into the final parsimonious model. Given that no patients who received IE valve surgery died, this variable was unable to converge in the final model and was excluded.

**Table 3:** Variables associated with all-cause, in-hospital mortality^a^

Characteristic	Unadjusted OR 95%CI	*P*-value	Adjusted OR 95%CI	*P*-value
Elevated troponin	6.1 (2.9–12.9)	< 0.001	7.3 (3.3–15.9)	< 0.001
Severe Sepsis/Shock	9.8 (4.3–22.3)	< 0.001	Not Tested	–
Acute renal failure	7.2 (3.3–16.3)	< 0.001	Not Tested	–
Septic emboli	3.0 (1.3–6.6)	0.005	Not Tested	–
Left-sided IE	2.6 (1.3–5.4)	0.006	Not Tested	–
Atrial Fibrillation	2.2 (0.89–5.7)	0.09	2.7 (0.97–7.8)	0.06
Age greater than 40 years	1.9 (0.95–4.1)	0.07	2.0 (0.9–4.6)	0.09
MRSA IE	1.8 (0.9–3.7)	0.12	2.6 (1.2–5.8)	0.02
Heart failure	0.97 (0.42–2.3)	0.95	Not Tested	–
Valve surgery	0.85 (0.81–0.9)	0.01	Not Tested	–

^a^Hosmer-Lemeshow Goodness-of-fit test: Chi-square, 1.6; *P*-value, 0.90

Given the co-linearity observed between renal dysfunction elevated troponin values, a sub-group analysis of patients without acute renal failure was performed. The results of the bivariate and multivariable models were similar in nature to the original analysis, as troponin values remained significantly associated with in-hospital mortality (adjOR 3.3; 95%CI, 1.2–9.0).

## Discussion

Our study found that patients with elevated troponin values and MRSA IE were associated with increased in hospital mortality. The overall observed inpatient mortality rate was 12%, which is lower than published literature [3]; one explanation is the large number of patients with mainly associated right-sided IE (65%).

In our study, which is to our knowledge the largest to-date evaluating the role of troponin as a predictor of mortality, patients presenting with an elevated troponin had an adjusted OR of 2.5 of death before discharge (95% CI 1.1–5.6), confirming previous published reports describing an inverse relationship between survival and increased troponin levels, and indicating the importance of obtaining troponin levels for admitted patients with IE to help stratify their mortality risk [9, 10].

Few previous reports with small sample sizes described elevated troponin as surrogate for increased likelihood of death in patients with IE. Purcell et al. reported increased troponin levels in 65% of 51 patients with IE studied, the study also described an association with central nervous system involvement and death [12]. Tsenovoy et al. reported increased troponin I levels in 57% of 62 patients with IE, with in-hospital mortality or valve replacement occurring in 18 patients (51%) of the increased troponin arm; these findings were also demonstrated by Stancoven and colleagues [13, 14]. Gucuk et al. found that troponin levels could also predict one-year survival rates in IE, with 41% mortality rate during follow up in patients with higher troponin levels during initial hospitalization [9]. There has been postulation that an elevated troponin level indicates myocardial involvement of infection. This would likely indicate a more significant infection compared to only endocardium or valvular involvement. Another possibility is that elevated troponins are associated with more severe sepsis, such as that associated with demand ischemia [9, 12].

Other predictors of inpatient mortality described in our report included patients older than 40 years of age, severe sepsis or shock on presentation; these predictors are similar to previously described literature emphasizing the importance of initial presentation with multi-organ dysfunction and risk of systemic embolization [15–20]. In a study by Ferrera et al., IE patients presenting with atrial fibrillation had higher in-hospital mortality independently from other relevant clinical variables [21]. Our findings are similar, but ultimately atrial fibrillation was not associated with in-hospital mortality in the final parsimonious model.

We did find an association between different organisms including MRSA and mortality, some reports suggested increased mortality with *S. aureus* [22, 23], and other reports did not [24]. The PWID population remains of high-interest due to increasing incidence of IE and significant morbidity and long-term complication [5].

The limitations of our study included that it is a single center study with high percentage of PWID, and the results may not be reflective of the general population. However, given the lasting negative effects of the opioid epidemic in the United States, we feel these data likely represent a common patient population particularly throughout the southern United States [25, 26]. Another limitation was in the retrospective data collection through the electronic medical record, limiting the ability to gather additional information on each patient especially pertaining to drug use and/or other variables that may be associated with in-patient mortality. However, we attempted to correct for confounding variables through multivariable logistic regression methods. We did not collect information on treatment regimens and need for surgery during hospitalization, and patients leaving against medical advice and loss to follow up data.

## Conclusion

The incidence of inpatient mortality was higher in patients with IE and elevated cardiac troponin I levels compared to patients with normal levels; cardiac troponin I may have potential as a prognostic marker in IE.

## Data Availability

The datasets analyzed during the current study are not publicly available due to protection of the patients’ privacy, but are available from the corresponding author on reasonable request.

## Abbreviations

HIV/AIDS: Human immunodeficiency virus/acquired immune deficiency
IE: Infective endocarditis
LAMA: Left against medical advice
MRSA: Methicillin-resistant *Staphylococcus aureus*
PWID: People who inject drugs
